# Barriers and opportunities for cleanliness of shared sanitation facilities in low-income settlements in Kenya

**DOI:** 10.1186/s12889-020-09768-1

**Published:** 2020-10-31

**Authors:** Sheillah N. Simiyu, Raphael M. Kweyu, Prince Antwi-Agyei, Kwaku A. Adjei

**Affiliations:** 1 African Population and Health Research Center, P.O Box 10787-00100, Nairobi, Kenya; 2 Kenyatta University, P.O. Box 43844-00100, Nairobi, Kenya; 3 University of Energy and Natural Resources, P.O. Box 214, Sunyani, Ghana; 4 Kwame Nkrumah University of Science and Technology, Private Mail Bag, University Post Office, Kumasi, Ghana

**Keywords:** Behaviour change, COM-B, Kisumu, Low-income settlements, Landlords, Shared sanitation cleanliness, Tenants

## Abstract

**Background:**

The sharing of sanitation facilities is a common practice in low-income areas in sub-Saharan Africa. However, shared sanitation is currently categorized as a limited sanitation service, and may therefore not count towards meeting the global goals. These shared facilities are often the only option available for most residents in low-income settlements, and improving their cleanliness and overall management is key to reducing open defecation and risk of disease. This study sought to investigate barriers and opportunities for improved cleanliness of shared sanitation facilities in low-income settlements of Kisumu city, Kenya.

**Methods:**

Thirty-nine in-depth interviews and 11 focus group discussions were held with residents – mainly tenants and landlords – of a low-income settlement in Kisumu. Analysis followed a thematic approach to define the problem, specify the target behaviour and identify the changes needed.

**Results:**

Sanitation facilities were mainly pit latrines, typically shared among landlords and tenants. Participants singled out behavioural (poor use of the shared toilets) and social (lack of cooperation in cleaning) challenges that led to unclean shared toilets. Available opportunities for improvement included instituting clear cleaning plans, improving communication among users, and enhanced problem-solving mechanisms between landlords and tenants. These approaches could form the basis for designing intervention strategies for improving the cleanliness of shared sanitation facilities.

**Conclusion:**

The results highlight the need to focus on social aspects for improvement of cleanliness in shared sanitation facilities in low-income settlements. Through a social approach, shared sanitation facilities can be managed appropriately to provide the millions of low-income residents in Kenya an opportunity to access sanitation. This study provides further evidence on approaches for improved management of shared sanitation facilities in line with the World Health Organization’s (WHO) Joint Monitoring Program’s (JMP) recommendation for high quality shared facilities.

## Background

Since the 1960s, the urban population worldwide has risen steadily, leading to the urbanisation of poverty, inequality, and the expansion of low-income settlements. In Africa, approximately 62–70% of the urban population lives in low-income settlements [1]. These settlements are faced with challenges such as inadequate water and sanitation services. Due to these inadequacies, most households share sanitation facilities with other households, facilitating access to sanitation for residents who would otherwise lack these services.

Sanitation facilities that are shared by two or more households are classified as ‘limited’ sanitation service by the Joint Monitoring Program (JMP) of the World Health Organisation (WHO) and the United Nations Children’s Fund (UNICEF) [2, 3]. This classification is mainly due to increased health risks of exposure to faecal matter, and human rights concerns relating to dignity and safety [2, 3]. This classification also implies that any improvements to shared facilities may not count towards meeting global sustainable development goals, which may result in investments and improvements not being directed towards users of the shared facilities [4].

Studies have confirmed that the number of users, the relationship among users, and lack of cooperation contribute to the low levels of cleanliness of shared sanitation facilities [5–10]. Unfortunately, since shared sanitation facilities are often the only option available for most residents in low-income areas, it is feared that users may revert to open defecation practices if the facilities are inaccessible or unclean [9]. Conversely, it is acknowledged that ‘high quality’ shared sanitation facilities may be the best interim solution in low-income settlements [2, 3], and thus, interventions to improve the cleanliness of these sanitation facilities are needed.

Unfortunately, studies focusing on such interventions are few, thereby limiting the available options to learn from in developing relevant interventions. Emerging evidence from Bangladesh [11] and Uganda [12] for example, has demonstrated the usefulness of deploying communication and behaviour change theory in addressing the cleanliness of shared sanitation facilities.

Design of such interventions requires an understanding of the prevailing conditions within a country, more so in Africa where rates of shared sanitation facilities are the highest globally [2] and where the literature is devoid of evidence from such interventions. In Kenya for example, it is estimated that 22% of the population uses limited (shared) sanitation, and 44% of urban residents - especially those in low-income areas - share sanitation facilities [3]. These shared sanitation facilities act as a solution to the lack of household sanitation services in the settlements, therefore their proper overall management will facilitate sustained use and contribute to a reduction in open defecation. Interventions to improve cleanliness of shared sanitation facilities in Kenya’s urban areas, however, are few. Additionally, interactions among individuals (such as landlords and tenants) in ensuring the cleanliness of these shared toilets is an important consideration especially since tenants are the majority of residents in these settlements [13]. This study therefore aimed at investigating the factors that enable or hinder cleanliness of shared sanitation facilities in an urban low-income settlement in Kenya in order to design and test intervention strategies for improved cleanliness of shared sanitation facilities.

### Theoretical approach

The study was guided by the Behaviour Change Wheel (BCW) approach to characterize and design behaviour change interventions [14]. The BCW is a framework developed from multiple models of health behaviour, and enables the systematic development of interventions for supporting behaviour change [14]. The wheel has been applied to understand behavioural determinants and design interventions aimed at instilling good hygiene habits in schools in Uganda [15], understanding and development of caregiver hygiene behaviours [16, 17] and other health related interventions [18–24].

**Fig. 1:**
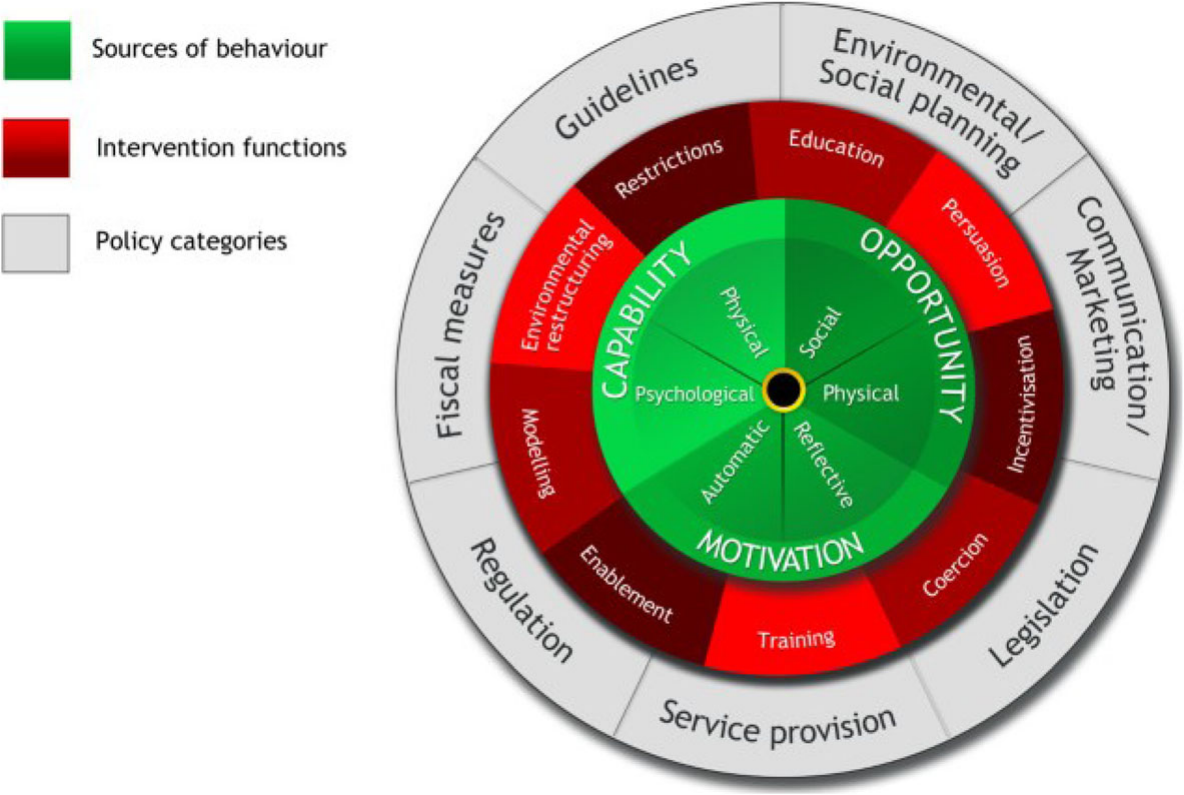
The Behaviour Change Wheel. Source: Michie et al., 2011 [14]

The wheel (Fig. 1) is divided into three rings: (1) the inner ring aims at understanding behaviour and identifying what needs to change; (2) the middle ring identifies interventions that are likely to initiate behaviour change, such as training, education, persuasion, coercion and restrictions; and (3) the outer ring identifies the relevant policy categories that can support the change, such as legislation, service provision, regulation, fiscal measures and guidelines.

The inner ring is underpinned by the COM-B (Capabilities, Opportunities and Motivations that enable or inhibit behaviour) model that outlines necessary conditions for a behaviour to occur. Capability is an individual’s psychological and physical capacity and entails having skills and knowledge to engage in an activity. Opportunities are factors in the physical and social environment that enable or hinder behaviour, while motivations are reflective and automatic brain processes that energize and direct behaviour [14]. Automatic motivations involve emotions and impulses arising from learning and/or innate disposition, and reflective motivations are brain processes that involve evaluations and plans [14].

The COM-B model which guides behaviour identification entails four steps: (i) problem definition; (ii) selection of the target behaviour(s); (iii) specification of target behaviour; and (iv) identification of what needs to change. This paper is part of a study that aims at improved management of shared sanitation facilities in low-income settlements in Kenya and Ghana. The paper focuses on these four stages of the inner ring in order to understand barriers to cleanliness of shared sanitation, which would then guide subsequent stages including development and testing of interventions, as well as identification of relevant policy categories for improving shared sanitation in the two countries. Results of testing interventions for behaviour change and policy recommendations will be presented in subsequent manuscripts.

## Methods

### Study area

This study was conducted in the low-income settlements of Kisumu city in Kenya. Kisumu city is located in Kisumu County, and is the third largest city in Kenya. The city has an estimated population of 500,000 according to the 2019 population and housing census [25]. Sixty percent of this population lives in low-income areas characterised by overcrowding, poor housing, and lack of basic services [26]. Land ownership in the settlements is mainly freehold, with families handing down land to successive generations and/or to spouses [27]. Over the years, landowners have constructed rental structures to accommodate the rising demand for rental housing. These housing structures are usually organised in compounds (often called plots) that comprise several single adjoining unit houses occupied by different households (often unrelated), most of whom are tenants. Some landlords reside within the compound while some live away [27]. Basic services such as water, sanitation and solid waste disposal are shared among compound members, and landlords are required to ensure that these services are provided. The relationship between landlords and tenants varies. Some landlords and tenants have a cordial relationship, other landlords have a general feeling that tenants are uncooperative, and in other cases, there is a general understanding among landlords and tenants, especially with regards to payment of rent [27–29].

It is estimated that approximately 65% of residents in the settlements use improved sanitation technologies that are often shared [30]. Studies have highlighted the nature and consequences of sharing, indicating that most of the shared facilities are soiled with faeces [7, 30]. These studies, however, did not examine the barriers, facilitators, and opportunities for improvement of the cleanliness of these shared facilities, nor did they test any interventions. Our study advances this research by identifying improvement opportunities and testing appropriate interventions. We, however, focus at the containment/household level by examining cleaning practices of shared sanitation facilities.

### Approach and participant selection

A case study approach was adopted through the use of qualitative data collection methods. This approach is appropriate as it is used to study complex, real life social settings and interactions among the study subjects (who in this case were users of shared sanitation) to establish causal relationships [31, 32]. Additionally, a case study design allows in-depth exploration through multiple methods [31, 32]. The study was conducted in Nyalenda A, an informal settlement in Kisumu which is divided into four clusters (called units) namely, Central, Western, Kamakowa and Dago. A Community Health Volunteer (CHV) was identified from each of the 4 units, and each CHV worked with field assistants to identify the boundaries of the units. This identification was necessary to ensure that compounds were selected from all the units, and that CHVs only worked within their respective units. Each CHV was paired with a research assistant. CHVs and research assistants moved from one end of the unit to the other, skipping at least three compounds, and ensuring that the selected compounds had not been selected during an earlier quantitative phase. Compounds were purposely selected if there was a sanitation facility that was shared between two or more households. Sharing of sanitation facilities is common within the settlement, and such compounds were easily singled out by identifying the toilet structure within or near the compound. Additionally, CHVs were familiar with their units and they easily identified the toilets. To verify that the toilets were indeed shared, the team identified a member from the compound to confirm whether the toilets were shared, as well as the type of residents in the compound (e.g. if the landlord was a resident, or whether there was a caretaker).

Where applicable (and if they were available), resident landlords were first approached in order to obtain the necessary permission to carry out interviews within the selected compound. Once permission was obtained, the landlords were selected and interviewed (if they were willing) to ensure that they were included in the sample since majority of residents in the settlements are tenants. If a landlord was not available, the caretaker was approached and interviewed, and if unavailable, one tenant household was randomly selected. Participants had to be adults (defined as being at least 18 years) who had lived in the compound for at least 1 month and were users of the shared sanitation facility within their compound. These criteria ensured that minors (below 18 years) were not interviewed, and that selected respondents were familiar with the issues around their shared toilets. All the potential participants approached consented and participated in the study.

### Data collection

Data was collected through In-depth Interviews (IDIs) and Focus Group Discussions (FGDs). The interviews were based on an in-depth interview guide that had questions on sharing, usage, cleaning and maintenance practices of the shared sanitation facilities within the compound. Participants were for instance asked to describe the cleanliness of their toilets, the cleaning practices (who, how and what is used for cleaning), challenges experienced in sharing the toilets, and recommendations for improvement. Participants were allowed to choose the language of communication, and as such some interviews were conducted in the local language (Dholuo) and others in Swahili. Information was captured through notes and by an audio recorder, with interviews going on for 20–40 min. Interviews were led by the research assistants and the researchers, and they continued until the point when new information was not forthcoming [33]. This point of saturation was determined through continued interaction with information during data collection. The interaction enabled the identification of issues that required further probing, as well as individuals who needed to be interviewed. Saturation was determined to have been achieved when landlords and tenants had been included in the sample, and when all the questions were being answered in the same manner without any new information.

Data was also collected through FGDs to validate the information obtained from the IDIs, to understand social interactions and norms that influence the cleaning of shared sanitation, as well as to identify opportunities for improvement from a social level. Based on information from the previous data collection stage, it was deemed necessary to have FGDs based on residence types, for example, with participants from compounds with resident landlords, or with participants from compounds without a resident landlord. Besides validation of the findings, this classification enabled further investigation of issues such as roles of landlords and tenants, the relationship between landlords and tenants especially when they live in the same compound, and opportunities available for landlords and tenants to participate in management of their shared facilities. FGD participants were therefore selected if they were from such compounds, if they were users of shared sanitation facilities within their compounds, and if they had not been selected for the IDIs. CHVs and the research assistants again walked through the units to identify and select FGD participants. CHVs and research assistants selected a certain number of participants from their unit (for example 3–4 participants of each gender), to ensure that participants were from all the units and that each FGD had an adequate number of participants. These participants were identified a day or two before the actual meeting.

All FGDs consisted of 8–12 participants and they were held at a central location that was easily accessible by residents. For each category of discussants, male and female participants held their discussions separately in order to distinguish the roles played by men and women in cleaning of the shared sanitation facilities. Each FGD had a moderator, a note taker and an observer, with the research assistants moderating and taking notes, while the researchers served as observers. The moderator was guided by an FGD guide with questions on sharing, cleaning and maintenance practices, as well as challenges and opportunities for improvement. Again, participants agreed on the language to be used during the discussions at the start of the meeting, with some group discussions being conducted in Dholuo and others in Swahili. Proceedings were captured in notebooks and as well as via a recording device with discussions going on for 60–90 min. The total number of FGDs was not determined a priori, rather emphasis was placed on ensuring that FGDs were held with all people involved in overall management of shared sanitation facilities. Similar to the IDIs, saturation was achieved when FGDs had been held with the different categories, and when new information was not forthcoming. An additional two FGDs were then held with landlords and tenants as participants in the same group to verify that all information from the previous discussions had been captured. The interview and group discussion guides developed for this study have been published elsewhere [34].

### Data quality control

Research assistants were recruited based on previous experience in conducting qualitative research and their understanding of the local language. Each of the assistants had a minimum of a basic university degree, at least 2 years’ experience in qualitative data collection, and had worked with the researchers before in previous studies. These assistants were thoroughly trained for 3 days before data collection began. During the training, the questions were translated to the local dialects which were counterchecked by three independent individuals who understood the local language. Role play was used during training to ensure that questions were asked in the right way, and to demonstrate the principles of good research practice such as probing and not asking leading questions. The tools were pre-tested to ensure that the research assistants understood the questions and asked them correctly, as well as to confirm that the participants understood the questions.

Meetings were held with CHVs and the research team to discuss good research practices such as selection of participants for the study. Debrief sessions were held at the end of each day to evaluate progress and the data collected, to identify issues that needed further probing and follow up (deviant cases), and to adjust accordingly. Researchers worked with research assistants to ensure that data collection and probing were done sufficiently. Researchers also observed and noted the respondents’ body language. All the recordings were transferred to protected computers and backed up on an online storage platform.

### Data management and analysis

Interaction with data began during data collection. If the information provided was not sufficient, research assistants probed further for more information. All the recordings were transcribed verbatim onto Microsoft Word and later translated to English. The translated documents were read by a third party to verify that information and meaning was not lost during the translation. The transcripts were read and re-read to understand the ‘story’ and tease out the emergent themes. The transcripts were then transferred to ATLAS.ti where analysis followed a thematic approach. The transcripts were first labelled (coded) based on emergent words or issues, e.g. dirty/clean toilet, cleaning, etc. As the analysis continued, new codes emerged and transcripts were read again to ensure that the new codes were captured in the analysis. The codes were then merged into categories such as use, cleaning practices, users, etc. Some categories were later merged into themes based on the theoretical model. During the analysis, any unusual information from the transcripts (deviant cases) was identified and the transcript was read again to understand the positive or negative deviation, and/or the reasons for the deviation. Finally, some transcripts were read by another researcher to confirm their validity and ensure that all the information had been coded adequately.

## Results

Thirty-nine in-depth interviews and 11 FGDs were held. Twenty-two of the IDI participants were landlords and 17 were tenants. The number and participants of the FGDs are summarised in Table 1. Results have been presented according to the steps of the COM-B model, i.e. problem definition; selection and specification of target behaviour; and understanding what needs to change.

**Table 1:** Summary of FDGs conducted

Category of FGD participants	No of FGDs
Resident male landlords who had tenants on their compounds	1
Resident female landlords who had tenants on their compounds	2
Male tenants from compounds with resident landlords	1
Female tenants from compounds with resident landlords	2
Male tenants from compounds with absentee landlords	1
Female tenants from compounds with absentee landlords	1
Caretakers	1
Mixed group of resident male landlords and male tenants	1
Mixed group of resident female landlords and female tenants	1
**Total FGDs**	**11**

### Characteristics of sharing

#### Living arrangements and sharing

All the sanitation facilities were pit latrines shared among households who lived in compounds occupied by tenants only, by tenants and landlords, or by tenants and caretakers. Latrines were single cubicles or 2–3 cubicles over the same pit. The latrines were the squatting type with a drop hole, most had concrete slabs, and the superstructure was constructed using blocks, bricks or iron sheets. The cubicles were approximately 2 m wide and 2 m long, and none had waste receptacles inside. Resident landlords often constructed more than one cubicle and separated one of the cubicles for their own household use with tenants sharing the other cubicles.

#### Number of households and users

One cubicle was reportedly shared with a minimum of 4 and a maximum of 13 households. The actual number of users was dependent on the number of people in each household, which ranged from 3 to 8 members. It was also reported that other residents in the settlement, who were not from the compounds often used the toilets. These other users were from compounds without toilets or from compounds whose toilets were full. Whereas some participants mentioned the actual number of users, e.g. *“approximately twenty”* and *“we are thirty-four people”,* others often said “*we are many*” to describe the high number of users whose number was not known. Some landlords also admitted that users in their compounds were *‘so many* [they could not] *approximate the number* or *have never taken count’* (Participant in an FGD with female landlords).

### Problem definition

#### Shared sanitation cleanliness

Unclean toilets, high number of users, and high fill up rates of pit latrines were cited as the main challenges in sharing of sanitation facilities. Resident landlords or tenants who lived on compounds with resident landlords often reported that their toilets were clean, whereas tenants who lived on compounds with an absentee landlord often admitted that their toilets were unclean. Unclean toilets resulted from improper use or disposal of human faecal matter – often on the slab – which discouraged others from using the toilets. Improper usage also extended to other behavioural practices, e.g.:

“*There is a man who normally gets drunk and he vomits on the slab of the toilet.” (*Participant in an FGD with female tenants from a compound without resident landlord).

Compounds with children were more likely to have dirty toilets, especially if the toilets were not cleaned after the children soiled the toilets. Toilets were also dirty because other non-compound residents soiled the toilets. These users gained access to the compound toilets forcefully, by sneaking in without permission, or by befriending the compound residents. Participants noted that such users accessed the toilets when they [the toilets] were not locked. While participants suggested that locking the toilets was preferred to keep the non-compound users away, others admitted that sometimes the keys got lost or that locks were broken leaving the toilets easily accessible.

Participants complained of a high number of users per cubicle, some of whom were not willing to take part in cleaning the toilets. Some tenants, for example, were not willing to clean toilets because they felt it was not their responsibility.

*“…Some go as far as telling you that once he cleans the toilet then he/she might become sick* [if they clean the toilets]*.” (*Participant in an FGD with female tenants residing in compound without resident landlord).

As a result, quarrels and disagreements ensued among compound residents when users soiled the toilets but did not participate in cleaning.

Participants also disliked the foul odour from the dirty toilets and expressed fears that dirty toilets posed risks of disease spread especially to women and children, citing diseases like cholera and syphilis.

*“…It's easy for women and children to get infection because when they squat they collect dirt unlike us men who stand when we urinate”* (Participant in an FGD with male landlords).

The high number of users which led to queueing, and indiscriminate disposal of solid waste in the toilets, both of which led to rapid fill up of the pit latrines, were also disliked. Such solid waste included diapers, sanitary towels, pieces of clothing etc.

### Selection and specification of the target behaviour

Strategies for improving the cleanliness of shared toilets were mainly *behavioural* and *social* in nature. Resident landlords for instance admitted that they sometimes cleaned the toilet as it was not easy to identify individuals who had soiled the toilet, whereas in tenant-only compounds, tenants who were usually women with children, volunteered to clean the toilets. Nonetheless, tenants and landlords were categorical that all users ought to use the toilet in a proper manner and participate in cleaning.

*“People …should squat well when using the toilet…so that faeces go directly into the pit… what you have used [*anal cleansing material*] should go into the pit after finishing… then you lock the toilet’s door using a padlock, take clean water and soap and clean it*.” (IDI with female tenant).

Landlords and tenants admitted that the cleanliness of the toilets was related to the number of households/users, with some admitting that they were comfortable sharing with a small number of households. One landlord for instance, speaking about sharing with his tenants, admitted that *“They are few…I take them as my family, but if it was a large number of tenants, I would not be free because some tenants are tough headed*.” (Participant in an FGD with male landlords in Dago). A tenant also admitted that in addition to the smaller number, the households cooperated in cleaning of the shared facilities. Other individual level behavioural strategies that were suggested included cleaning the toilets with appropriate cleaning products (water, soap) and disinfectant, and avoiding the disposal of diapers and sanitary towels in the pit latrines. Individual social strategies entailed improving communication between landlords and tenants, among tenants themselves, and commending individuals who cleaned the toilets.

Participants further pointed out that cleanliness of the toilets also depended on strategies implemented as a group or at the compound level, such as restricting access for other non-compound users, cooperation with compound residents in purchasing cleaning items, and establishing cleaning schedules that included all households. A female tenant in an FGD for instance reported that the reason why their toilet was clean was, *“We have got a gate and whenever* [outsiders] *want to access the toilet then they have to ask…. we contribute money for buying brooms since we scrub the toilet with a broom…. we are aware of how we are cleaning… each and every person is aware of his/her week of cleaning….”* (Participant in an FGD with female tenants residing on compound without resident landlord). Proper maintenance of the shared toilets was attributed to agreements and cooperation among users in the compound, with a landlady stating that “*When there is no agreement* [on cleanliness]*, the toilets will definitely not be clean*.” (IDI with landlady).

At the compound and/or group level, regular discussions among tenants and landlords were proposed, with discussions focusing on the maintenance of the shared toilets.

*“Landlords should partner with their tenants, for example, once in a month [*they should*] have a meeting to discuss the state of cleanliness, who cleans and who does not, and the number of users should be known”* (participant in an FGD with female tenants and landlords).

It was noted that such social strategies lead to open discussions among the households, the resolution of contentious issues, development of a united group of households, and an improved management plan. Participants in the landlords and tenants FGDs further suggested the use of community-based groups or individuals (such as a caretaker) who offer cleaning services for toilets within the informal settlement at a fee.

The need for education and creation of awareness was often highlighted, so that residents would understand the importance of keeping their toilets clean and the need to use appropriate cleaning materials (why shared toilets should be clean, and how they should be cleaned). Such strategies were proposed for all residents within the compounds (including children). Other proposed suggestions included penalties for individuals who did not participate in cleaning, continued monitoring, and provision of bins for the segregation of waste.

These results specified the aspects of any targeted behavioural intervention in terms of the individuals to be involved and the actions to be taken. Specification of these strategies would inform the designing and testing of interventions that can be taken up by the community members and supported by other individuals or interested parties.

### Understanding what needs to change

The third stage entailed identifying capabilities (psychological and physical), opportunities (physical and social), and motivations (automatic and reflective) that were enablers and barriers to cleanliness of shared sanitation facilities.

#### Psychological and physical capabilities

A general knowledge and understanding that shared toilets should be cleaned was noted, and because of this knowledge some participants reportedly cleaned the toilets before or after use. Some women, for example, understood the importance of, and their responsibility in cleaning the toilets before and after their children used the toilet.

#### Physical and social opportunities and barriers

Cleaning materials such as water, brooms, and detergent facilitated cleaning. These materials were bought by landlords, or by tenants who contributed money towards their purchase. Compounds that were fenced or had a gate restricted the entry of other users who might soil the toilets, while those without a gate or fence were more likely to have intruders and hence unclean toilets. Tenants in such compounds were less motivated to clean their toilets. Other forms of barriers included the use of padlocks which ensured that users from outside the compound did not gain access to the toilets. However, landlords highlighted that some tenants lost the keys, or that other users broke the padlocks in order to use the toilets.

With regards to social opportunities and barriers, resident landlords were strict about the cleanliness of the toilets, which ensured that the toilets were kept clean.

*“Our landlord has a stern rule that he does not want to find anyone destroying or misusing the toilet. He keeps quarrelling on this matter, and this has helped in maintaining the cleanliness of the toilet.*” (Participant in an FGD with female tenants residing on compounds without resident landlord).

Other compounds had strict rules about cleaning the toilets which were adhered to by all tenants. These rules were sometimes part of the ‘contract’ when a new tenant moved into the compound, and included mandatory cleaning by all tenants, often in the form of a cleaning schedule. The schedule was not always written, but was an informal rota understood and accepted by compound residents. A household’s turn to clean the toilet for example was determined by the housing order within the compound, or by the day of the week. Thus, it was common to hear that a household would clean after another household, or on a certain day of the week.

Both landlords and tenants further proposed that the rules should be mandatory and tenants who did not comply should be asked to vacate the compound. The cleaning schedule was often a reminder to the individuals themselves and to other compound members. Individuals cleaned the toilets when it was their turn, and if they forgot, their neighbours and/or landlords reminded them. Some landlords however, noted that the cleaning schedule was sometimes not effective because tenants did not adhere to the schedule.

It was interesting to note that in the two FGDs where landlords and tenants were participants, landlords confessed that tenants did not always clean the toilets, and as such they took on the responsibility either by cleaning the toilets themselves, tasking the caretakers to clean the toilets, or paying for the toilets to be cleaned by other individuals or community groups. For example, a landlord admitted that since the tenants did not adhere to cleaning, he had assigned the responsibility to the caretaker, noting that *“When I find out that the toilet is dirty, I ask my caretaker why the toilet is not clean…. that’s I why I have a caretaker …*” (IDI with Landlord). Tenants on the other hand, did not refute this admission, agreeing instead that landlords should be involved in cleaning the toilets.

Generally, FGD participants admitted that sometimes the cleaning schedule included the landlords and the tenants, and such arrangements were often agreed upon in meetings involving all compound members. A landlord for instance explained that he *called for a meeting and told his tenants that he would be responsible for* [buying] *the detergents, but they would be responsible for cleaning.* He went on to explain that this arrangement worked well because ‘*when tenants requested, he provided the detergent’.* He noted that this arrangement was beneficial as the tenants *‘took it as their responsibility.” (*Participant in an FGD with male landlords). Such compound meetings were also called for to resolve issues affecting the tenants. In an FGD among male tenants and landlords, a tenant commended another landlord saying *“In certain occasions he* [the landlord*] calls for meetings, writes letters to tenants telling them that on a certain date we have a meeting…. during the meeting, he will communicate if there was a mistake… …corrects mistakes…if a child soils the latrine, he tells them that it’s the role of the parent to clean it*.”

Within tenant only compounds, tenants had established agreements among themselves about cleaning the toilets. Such social agreements and arrangements ensured that all tenants participated in cleaning. For example, a tenant noted that they had a cleaning schedule, but when one was not available to clean the toilet on their appointed day, the individual made arrangements to clean the toilet the next day. This tenant emphasized that such agreements ensured that all members participated in cleaning.

Results further highlighted the different roles played by men and women, which were often agreed on at the compound level. Typically, men bought the cleaning materials while women cleaned the toilets.

#### Automatic and reflective motivations

Participants admitted that they cleaned the toilets because they wanted to be comfortable when using the toilet, while others cleaned because of the ‘urgent’ need to use the toilet.

*“I maintain the cleanliness because the toilet is a room where you would wish to go and feel comfortable, so it should be clean… you are like someone in jail when it is dirty because you cannot be comfortable in there.” (*IDI with landlady).

Dirty toilets discouraged use, and while some individuals cleaned the toilets before use, other users were less motivated to clean dirty toilets, and so used the toilets without cleaning.

The quality and structure of the toilet motivated or discouraged cleaning, e.g. toilets whose superstructure did not offer privacy, toilets whose slab was wooden, or toilets that were full. A tenant, when asked who cleaned the toilet, responded, “*Nobody… it is not in a good state…people just go for the sake of going… the toilet is almost full and it can cave in any time.”* (IDI with tenant).

Finally, some households cleaned the toilets in order to prevent the foul smell from infiltrating into their houses, especially when the houses were next to the toilets.

Reflective motivations were less evident in this study. The main finding was that women cleaned toilets because of the fear of contracting diseases or because of the fear of their children contracting diseases.

These capabilities, opportunities and motivations have been summarised in Table 2.

**Table 2:** Capability, opportunity and motivation enablers (+) and barriers (−) and possible intervention strategies for cleaning of shared sanitation facilities in low income settlements of Kisumu, Kenya

	Enablers (+) and barriers (−)	Possible intervention strategies
**Capabilities** (Individual’s psychological and physical capacities)	• An awareness that toilets need to be cleaned (+) • Awareness that toilets should be cleaned after being soiled by children (+)	• Need to increase awareness on use and cleanliness of shared toilets.
**Physical and social opportunities and barriers** (Factors in the physical and social environment that enable or hinder behaviour)	• Availability of cleaning aids e.g. water, detergents, disinfectant, and broom/brush (+) • Availability of individuals who clean, e.g. caretakers and youth groups (+) • Non-compound members who use and soil the toilets (−) • A stern/strict/firm resident landlord ensured cleaning is done (+) • A cleaning schedule in some compounds enhanced cleaning (+) • Cleaning rules that were adhered to by all users (+) • Landlords and tenants reminded other users to clean (+) • Responsibilities were shared among landlords and tenants (+) • Lack of or poor communication between tenants and landlords (−) • Poor structural quality of the toilets discouraged cleaning (−) • Children in the compound soiled toilets (−)	• Provision of cleaning aids • A barrier e.g. fence/gate to the compound. • Locking of toilets. • Defined and agreed upon cleaning schedule between landlords and tenants, or among tenants • Sharing responsibilities between men and women. • Improving relationships among compound members • Involving all users in cleaning • Communication and problem solving mechanisms among tenants, and between tenants and landlords • Improving on structural qualities of the shared toilets, e.g. emptying, and improving the superstructure
**Motivations** (Automatic and reflective brain processes that energize and direct behaviour)	• Users desire to be comfortable when using toilets (+) • Users clean toilets because it is the right thing to do (+) • Users are demotivated to clean dirty toilets (−) • Users clean shared toilets because of living next to the toilet (to avoid the smell) (+) • Users are demotivated to clean when cooperation was lacking (−) • Demotivation because of poor quality toilets (−) • Women feared contracting diseases (+) • Women feared their children contracting diseases (+)	• Enhance the motivation to clean shared toilets • Discourage poor use of toilets • Enhance the benefits of clean toilets and the disadvantages of dirty toilets

## Discussion

Sharing of sanitation is a common practice in the low-income settlements of Kisumu city in Kenya, with the dominant sanitation technology being pit latrines. Some of the toilets were reportedly clean while others were dirty mainly because they were not cleaned regularly. The main users of these toilets were individuals within the compound, and in other instances, individuals who were not resident in the compound. Social and physical opportunities for improving the cleanliness of shared toilets were most evident, with participants acknowledging that positive relationships, communication and cooperation among users, mutually established cleaning plans, and the quality of the toilet influenced the cleanliness of shared toilets.

With regards to problem identification and specification of behaviours that need to change, the study has highlighted that the reason why shared toilets were not clean was mainly because users did not clean the toilets. The lack of interest in cleaning shared toilets may be related to a general feeling of lack of ownership for shared toilets, especially among tenants, akin to resources that are used in common [7, 35]. The low involvement of tenants may also be because these tenants are temporary residents in the settlements who may move to other areas within or outside the settlements [28, 36]. However, results show that individual tenants and resident landlords often cleaned the toilets, driven by a sense of ownership, the desire to be comfortable when using a toilet, or the need to protect oneself or younger children from using dirty toilets. Resident landlords influence the cleanliness and overall quality of shared toilets by monitoring cleanliness, instituting rules of use and discussing with tenants matters related to the use of and cleanliness of shared toilets [28, 37]. Tenants in compounds with absentee landlords also ensured that their shared toilets were clean by having agreed upon standards of practice that guided the day to day management of toilets and spelt out the roles of users, monitoring systems, and frequency of cleaning.

Our study has highlighted that strategies to improve the cleanliness of shared sanitation should focus on social dynamics including the role played by social cohesion, improved communication, and social relationships. Enhancing these social aspects leads to improved maintenance plans (e.g. cleaning on behalf of another), joint resolution of sanitation related issues, combined efforts towards improved maintenance (e.g. joint purchase of cleaning materials) and better relationships among the compound members. Evidently, these aspects do not only lead to improved health outcomes, but also to additional social benefits (e.g. better relationships, less conflicts) some of which may not be easily quantified in the short term. Similarly other sanitation studies have highlighted indirect benefits such as psychological well-being as a result of improved sanitation [38]. The uptake of these strategies, however, will be influenced by the relationships between landlords and tenants, and among the tenants as users. Whereas landlords may have ‘control’ over their property, including the sanitation facilities, tenants also have a role to play in sustainable use of the shared facilities. Improved social relationships between landlords and tenants will, therefore, enhance adequate overall management of shared sanitation facilities.

As a way forward, and in line with the theory guiding this work, we note that strategies for improvement of shared sanitation facilities should focus on restrictions (using rules to reduce poor use or increase opportunities for proper use of shared sanitation), environmental restructuring (changing the physical or social context), enablement (increasing opportunities or reducing barriers to improved sanitation), increasing education and training, as well as improving the technological aspects of the sanitation facilities [14, 39]. Education and training may highlight the opportunities (including the social opportunities) lost if proper practices are not followed, e.g. the lack of privacy, risks of infection to children and women, quarrels in the compound; as well as the benefits of improved cleanliness of shared facilities, e.g. better social relations and quality of life, which would address the automatic and reflective motivations. These strategies should include individuals such as landlords and tenants (who may, for example, collectively define restrictions) at the individual and compound level, and community health volunteers and leaders (who may be instrumental in education interventions) at the compound and community level.

Policy categories may include creating guidelines that mandate the provision of shared sanitation (for example spelling out the minimum standards of provision and use of shared sanitation), fiscal measures (such as financial subsidies to landlords for sanitation provision), regulations (such as defining rules of use for shared sanitation including the roles of landlords and tenants), legislation (making laws where they are non-existent or improving the laws as needed), environmental/social planning especially in low-income areas, and service provision (such as cleaning services, and faecal waste provision services). In Kisumu County and more broadly in Kenya, these results imply that the current sanitation guidelines and legislation need to be cognisant of the services provided by shared sanitation facilities. It is, therefore, necessary to improve sanitation service provision in low-income settlements through the County level staff such as Public health officers and CHVs, and together with the community representatives. Additionally, sanitation guidelines should define the role played by shared sanitation towards meeting the national and global goals, in relation to the population being served. Although this may be a long-term process, our results are pointers to initial steps towards improving access to high quality sanitation facilities in low-income areas and improving the lives of the residents in these areas.

### Limitations

Our study mainly centred on cleaning practices that influence the continued use of shared facilities. We do realise however, that there are other aspects such as the safe management of faecal sludge from the shared facilities. These aspects of management beyond the containment facilities can be the subject of further studies. Additionally, our study presents findings from a low-income settlement in Kisumu city in Kenya. Whereas these findings may be applicable in other low-income settlements, we are aware that they may not explicitly apply in other areas whose conditions are not similar, and which have different social and cultural factors. Such variations may include suitability of sanitation technologies, land ownership and/or tenure, and the relationships between landlords and tenants. Nonetheless, in general, our approach can be applied in similar settings when designing and implementing behaviour change measures towards improved sanitation.

## Conclusion and implications

Using the Behaviour Change Wheel approach, this study has highlighted the barriers and opportunities for shared sanitation cleaning within a low-income settlement in Kisumu, Kenya. The results suggest that design and implementation of interventions should understand the local conditions and target the social aspects of shared sanitation. Such interventions should be cognisant of and be co-designed with users of the shared facilities. Education and sensitization on proper cleaning of shared facilities is critical, and it should target stakeholders including landlords and tenants (who are providers and users of sanitation facilities), community leaders (who are instrumental in community level interventions such as education and sensitization), and the local government (which defines and institutes policy measures and regulations). These results are the first step towards understanding the problem, and they will be used in subsequent steps of identifying and testing intervention strategies. From a global perspective, this study has provided evidence on approaches for the overall management of shared sanitation facilities, in line with the JMP’s recommendations for such facilities. Policy makers, practitioners and researchers can use this evidence to inform policy, design interventions and further the discussions on the classification of shared sanitation facilities.

## Data Availability

All data generated or analysed during this study are included in this published article. Transcripts are available from the author on reasonable request.

## Abbreviations

BCW: Behaviour Change Wheel
CHV: Community Health Volunteer
COM-B: Capabilities, Opportunities and Motivations for Behaviour
FGD: Focus Group Discussion
GLUK: Great Lakes University of Kisumu
IDI: In-Depth Interview
JMP: Joint Monitoring Program
NACOSTI: National Commission for Science, Technology and Innovation
WHO: World Health Organisation
